# Supplementary material for: Concurrent Heavy Metal Exposures and Idiopathic Dilated Cardiomyopathy: A Case-Control Study from the Katanga Mining Area of the Democratic Republic of Congo
Source: Int J Environ Res Public Health. 2021 May 6;18(9):4956. doi: 10.3390/ijerph18094956 (PMC8124897; doi:10.3390/ijerph18094956)
Supplement: Supplementary file 1 [file ijerph-18-04956-s001.zip › ijerph-1171681-supplementary.pdf]

Supplementary material

Table S1. Clinical features of subjects with dilated cardiomyopathy.

Category	Variable
**Comorbidity and other risk factor n(%)**	- Smoking in the past: 4(10)<br>- Alcohol consumption: 11(27)<br>- Clay consumption:16(39)<br>- Inactivity: 32 (78)
**Symptoms**	- Fatigue: 38(93)<br>- Breathlessness: 34(83)<br>- Dyspnea: 36 (88)<br>- Cough: 34(83)<br>- Orthopnea: 34 (83)<br>- Nocturnal dyspnea: 19 (46)<br>- Nycturia: 14 (34)<br>- Right hypochondre pain:5(12)<br>- Early satiety:35(85)
**Vital signs**	- PAS: 115±20 mmHg<br>- PAD: 83±16 mmHg<br>- Pulse: 98±13 /min<br>- SpO2: 96±5 %
**Signs**	- NYHA: II 12(29), III 23 (56), IV 6 (15)<br>- Jugular distension:13(32)<br>- Arrhythmia: 12(29)<br>- Murmur of MR: 30(73)<br>- Murmur of TR: 13(32)<br>- Tachypnea: 13(32)<br>- Pulmonary rales: 20(49)<br>- Hepatomegaly: 26(63)<br>- Ascites 4(10)<br>- Sacral edema: 27(66)<br>- Legs swelling: 29(71)<br>- Coldness of extremities: 1(2)
**Heart failure etiology n(%)**	- Dilated cardiomyopathy 33(80)<br>- Peripartum cardiomyopathy 8 (20)
**Echocardiographic findings & measurements**	- Thrombus 10(24)<br>- LVEF: 21±8 %<br>-LVIDD:6.4±0.6 cm<br>- LVEDV: 229±66 ml<br>- LVESV: 181 ± 55 ml<br>- LA volume index: 86±27 ml/m^2<br>- Mitral E/Ea average: 15±6<br>- TR Vmax 2.9±0.4 m/s
**12-lead ECG n(%)**	- LVH: 32 (78)<br>- LBBB: 3(7)<br>- Atrial fibrillation: 4(10)<br>- PAC:5 (12)<br>- PVC: 13 (32)
**24h holter ECG**	**- QTc : 587±125 ms**<br>**- long QT 17(52)**<br>**- Premature ventricular contractions**<br>Number: 202±297/h; 4852±7132/24h

	Connections : doublets 31(79)<br>Triplets 21(54)<br>Bigeminy 18(46)<br>Trigeminy 14(36)<br>Morphology: monomorphic 10(26)<br>Polymorphic 28(72)<br>Coupling: short 3(8)<br>Long 24(61)<br>Variable 12(31)<br>**-Ventricular tachycardia**<br>Non-sustained VT 20(51), 7±10 episodes/24h
**Treatment**	- Furosemide 39 (95)<br>- Beta-blocker 8 (20)<br>- ACE-i 35 (85)<br>- ARB 4(10)<br>- Spironolactone 9 (22)<br>- Isosorbide dinitrate 4(10)<br>- LMWH 2(5)<br>- Acenocoumarol 7 (17)
**Outcome**	- Inpatient 32 (78)<br>- Mean (SD) length of hospitalization 14±8 days<br>- Early mortality 4(10)

Table S2. Comparison of heart failure patients to controls according to demography, education level, and routine biology.

variables	Control (n=29)	HF patients (n=41)	p
Age, years	43±11	48±14	0.1121
Sex, n(%)			
Female	14(48)	20(49)	0.9668
Male	15(52)	21(51)	
Education's level			
Primary-to-secondary	12(41)	30(73)	**0.0075**
University and post-univ	17(59)	11(27)	
BMI, kg/m²	26.6±2.5	25.2±4.9	0.1414
Glycemia, mg/dl	88±18	108±37	**0.0042**
Total cholesterol, mg/dl	183±37	188±67	0.6965
HDL, mg/dl	46±9	40±19	*0.0723*
LDL, mg/dl	118±37	128±52	0.3708
Triglycerides, mg/dl	95±55	102±37	0.5387
Urea, mg/dl	26±7	53±30	**<0.0001**
Creatinine, mg/dl	0.9±0.2	1.2±0.4	**0.0005**
GFR, ml/min/1.73m²	85±18	66±21	**<0.0001**
Renal dysfunction, n(%)	2(7)	17(41)	**0.0014**
Uric acid, mg/dl	6.1±1.6	10.7±3.7	**<0.0001**
Total bilirubin, mg/dl	0.7±0.4	1.6±2.4	**0.0259**
Direct bilirubin, mg/dl	0.2±0.1	0.9±2.2	**0.0434**
AST, U/l	27±6	101±311	0.1351
ALT, U/l	29±13	84±148	**0.0225**
Calcium, mg/dl	8.7±0.3	8.4±0.6	**0.008**
Hypocalcemia, n(%)	3(10)	21(51)	**0.0004**
Magnesium, mg/dl	2.04±0.2	2.03±0.4	0.9167
Hypomagnesemia, n(%)	0(0)	6(15)	**0.0343**
Sodium, mEq/l	140.5±1.8	138.2±4.4	**0.0037**
Hyponatremia, n(%)	0(0)	10 (24)	**0.0039**
Potassium, mEq/l	4.2±0.4	3.7±0.7	**0.0018**
Hypokalemia, n(%)	1(3)	14(34)	**0.0024**
Chlorure, mEq/l	103.4±2.3	102.6±5.4	0.4198
C-reactive protein, mg/l	7±17	45±44	**<0.0001**
CRP, n(%)	2(7)	32(78)	**<0.0001**
White blood cell, x10³/µl	4.1±0.8	6.9±3.2	**<0.0001**
Lymphocytes, x10³/µl	1.9±0.5	1.8±1.1	0.3717
Monocytes, x10³/µl	0.4±0.1	0.7±0.3	**<0.0001**
Granulocytes, x10³/µl	1.8±0.5	4.5±2.9	**<0.0001**
Red blood cell, x10⁶/µl	5±0.7	5.2±0.6	0.3253
Hemoglobin, g/dl	14.2±2.3	13.6±1.4	0.1842
Hematocrit, %	40.7±6.7	38.5±5.7	0.1353
MCV, fL	81±9	76±7	**0.0080**
MCH, pg	28±4	27±3	**0.0188**
MCHC, g/dl	35±0.8	34±2	**0.0341**
RDW, %	13±3	16±3	**0.0001**
Platelets, x10³/µl	210±63	226±78	0.3490
MPV, fL	9.4±0.7	9.4±0.8	0.9530
PCT, %	0.19±0.06	0.21±0.07	0.2392
PDW, %	13.7±2.3	14.2±2.4	0.3718
INR	1.1±0.2	1.4±1.0	*0.0920*
Urinary Tract Infection	4(14)	12(29)	0.1064

Table S3. Correlations between blood (b) and urine (u) metal concentrations.

	**bAntimony**	**bArsenic**	**bCopper**	**bVanadium**	**bZinc**
uChromium	-0.22701	**0.40382**	**0.36284**	0.09298	-0.06249
	0.1056	**0.0030**	**0.0082**	0.5121	0.6598
uCopper	**-0.63067**	**0.42944**	**0.73235**	-0.28714	-0.41424
	**<.0001**	**0.0015**	**<.0001**	**0.0390**	**0.0023**
uZinc	**-0.55511**	**0.37591**	**0.59046**	-0.34853	**-0.35405**
	**<.0001**	**0.0060**	**<.0001**	**0.0113**	**0.0100**
uSelenium	-0.35934	**0.29941**	**0.46581**	0.00273	-0.08427
	**0.0089**	**0.0311**	**0.0005**	0.9847	0.5526
uCadmium	**-0.50295**	**0.40502**	**0.70435**	-0.17888	-0.31785
	**0.0001**	**0.0029**	**<.0001**	0.2045	**0.0217**
uAntimony	-0.39179	**0.43208**	**0.34748**	-0.30900	-0.29591
	**0.0041**	**0.0014**	**0.0116**	**0.0258**	**0.0332**
uThallium	-0.35644	0.18458	**0.42227**	-0.14857	-0.31281
	**0.0095**	0.1902	**0.0018**	0.2932	**0.0240**
uUranium	-0.29625	**0.37514**	**0.52591**	-0.10084	-0.35764
	**0.0330**	**0.0061**	**<.0001**	0.4769	**0.0092**

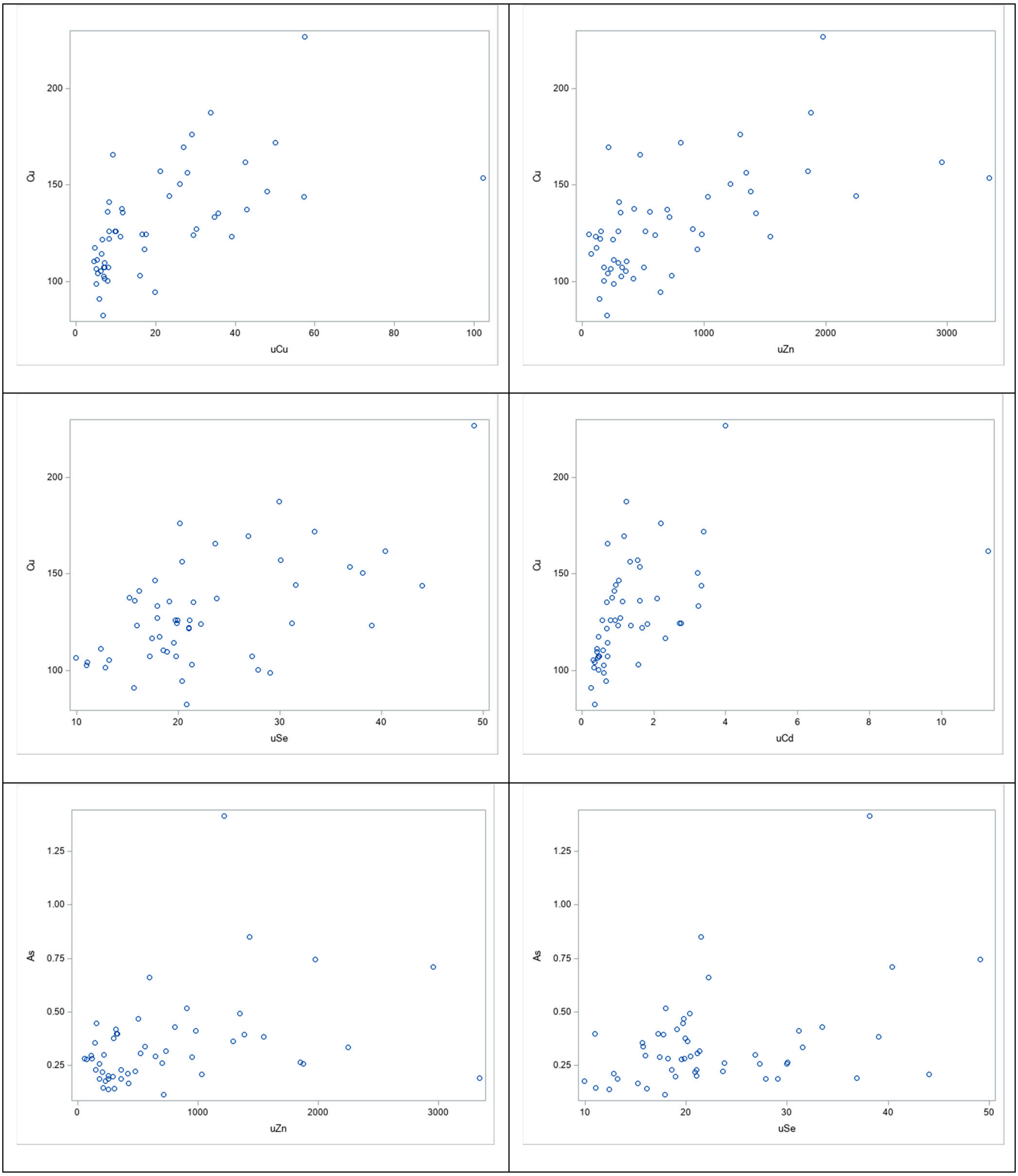

Figure S1. Illustrative correlations between concentrations of copper or arsenic in the blood and concentrations of zinc, selenium, cadmium, and copper in the urine.
